# Lower A1c among adolescents with lower perceived A1c goal: a cross-sectional survey

**DOI:** 10.1186/1687-9856-2013-17

**Published:** 2013-10-24

**Authors:** Scott A Clements, Matthew D Anger, Franziska K Bishop, Kim K McFann, Georgeanna J Klingensmith, David M Maahs, R Paul Wadwa

**Affiliations:** 1 Utah Diabetes & Endocrinology Center, University of Utah School of Medicine, 615 Arapeen Dr, Suite 100, Salt Lake City, UT 84108, USA; 2 University of Colorado School of Medicine, 13001 E 17th Place, N4223, Aurora, CO 80045, USA; 3 Barbara Davis Center for Childhood Diabetes, University of Colorado School of Medicine, 1775 Aurora Ct, A140, Aurora, CO 80045, USA; 4 University of Colorado School of Public Health, 13001 E 17th Place, B119, Aurora, CO 80045, USA

**Keywords:** Type 1 diabetes, Hemoglobin A1c, Adolescents

## Abstract

**Background:**

The International Society for Pediatric and Adolescent Diabetes (ISPAD) and the American Diabetes Association (ADA) have established a hemoglobin A1c (A1c) target of less than 7.5% for adolescents with type 1 diabetes (T1D). However, many adolescents are unaware of their A1c target, and little data exist on how knowledge of this A1c target affects the actual A1c they achieve. We sought to evaluate the relationship between awareness of the A1c target and the actual A1c achieved in adolescents with T1D.

**Methods:**

In a cohort of 240 adolescents with T1D age 13–19 years, we measured A1c and administered a questionnaire to assess their knowledge of the ISPAD guideline for A1c target.

**Results:**

Of the total cohort, 42 subjects (18%) had an A1c below target and 198 subjects (82%) had an A1c above target. Almost all subjects (98%) reported that they were told their A1c target by a healthcare provider, and most of those (88%) claimed to know their A1c target, but few (8%) were correct. More subjects with actual A1c below 7.5% thought their A1c goal was lower than the ISPAD target, compared to subjects with A1c above target (75% vs. 59%, p = 0.07), although this did not achieve statistical significance.

**Conclusion:**

In this cohort of adolescents with T1D, there was a trend toward a lower achieved A1c in those with a lower perceived A1c goal. Further studies should focus on identification of factors influencing an adolescent’s ability to achieve a lower A1c.

## Background

Hemoglobin A1c (A1c) is a widely used measure of glycemic control for patients with type 1 diabetes (T1D). Elevated A1c is associated with increased risk of developing microvascular and macrovascular complications, whereas an A1c less than 7.5% is associated with increased hypoglycemia [1-3]. Debate exists regarding the optimal A1c target for adolescents with T1D. Seeking to minimize long-term complications of diabetes, while also minimizing hypoglycemia, the International Society for Pediatric and Adolescent Diabetes (ISPAD) has established an A1c target of less than 7.5% for all children and adolescents with T1D [4]. However, scant data exist on how well adolescents can identify their A1c target or how knowledge of this A1c target relates to the actual A1c achieved by adolescents [5,6].

One determinant of achieving the A1c target in adolescents may be awareness of this target. If adolescents are aware of the A1c target, they may be more likely to work toward achieving that goal. If they are able to achieve a lower A1c, their risk of developing long-term complications of diabetes decreases. The purpose of this analysis was to determine the relationship between awareness of the A1c target and achievement of that target in a cohort of adolescents with T1D. We hypothesized that subjects who were aware of the A1c target would be more likely to achieve a lower A1c compared to those not aware of the target.

## Methods

### Participants

Data were obtained from a cohort of 240 subjects, age 13–19 years with T1D for a minimum of 5 years, from April 2008 through June 2010 [7]. Each subject was managed by a pediatric endocrinologist and the diabetes team at the Barbara Davis Center for Childhood Diabetes. There were an additional 60 subjects in the cohort age 12 years who were excluded from this analysis due to potential confusion between American Diabetes Association (<8%) and ISPAD (<7.5%) A1c targets for this age. Study participants with T1D were diagnosed by islet cell antibody and/or by provider clinical diagnosis. The study was approved by the Colorado Multiple Institution Review Board, and informed consent and assent (for subjects <18 years) were obtained from all subjects.

All subjects were followed in a pediatric diabetes subspecialty clinic by a team including pediatric endocrinologists, nurses, dieticians, and social workers. Subjects were generally seen every 3 months, with an A1c obtained at every visit. Diabetes providers in general review the target A1c of less than 7.5% with patients and families at every visit. On rare occasions, a provider may have suggested a higher A1c goal to avoid hypoglycemia or as a step towards better glycemic control in an adolescent in poor control, but these situations do not occur frequently.

### Study visit

All subjects fasted overnight (≥8 hours). Medical history was obtained with standardized questionnaires, including method of insulin administration (injections versus insulin pump). As part of a survey regarding cardiovascular health, subjects were asked questions about awareness of their A1c target. Blood pressure measurements were obtained after subjects had been laying supine for a minimum of 5 minutes. Height was measured to the nearest 0.1 cm with shoes removed using a wall-mounted stadiometer, and weight was measured to the nearest 0.1 kg using a Detecto scale. Tanner Stage was assessed by a pediatric endocrinologist, except in 29 subjects who refused assessment of pubertal status by a provider.

### Laboratory assays

A1c was measured on the DCA Advantage by Siemens (Princeton, New Jersey) at the Children’s Hospital Colorado main clinical lab. Total cholesterol, high-density lipoprotein cholesterol (HDL-c), and triglycerides were performed in the Clinical Translational Research Core (CTRC) lab using an Olympus AU400e Chemistry (Olympus, Brea, California). Low-density lipoprotein cholesterol (LDL-c) was calculated using the Friedwald formula.

### Categorization

The A1c target recommended by ISPAD of less than 7.5% for adolescents with T1D was used as the correct target. Those subjects who stated they knew their A1c target were asked to identify that target. Subjects were initially divided into 3 categories based on their responses: stated A1c target was correct, stated A1c target was below the ISPAD recommendation, and stated A1c target was above the ISPAD recommendation. However, some subjects stated an A1c range as the target, rather than a discrete number. If their stated A1c range was below the ISPAD recommendation of less than 7.5% (e.g. 6–7%), then they were included with the group whose stated A1c target was below the ISPAD recommendation. If their stated A1c range was above the ISPAD recommendation of less than 7.5% (e.g. 8–10%), then they were included with the group whose stated A1c target was above the ISPAD recommendation. If their stated A1c range included the ISPAD recommendation of less than 7.5% (e.g. 7–8%), then their responses were considered as a fourth category and were analyzed separately.

### Statistical analysis

Continuous variables were checked for the distributional assumption of normality. Because the majority of variables exhibited a skewed distribution, Wilcoxon Sign Rank test was used to test for differences between those who achieved their A1c target and those who did not. Chi-square test of independence or Fisher’s exact tests were used to test differences among categorical variables. Fisher’s exact test was used when there were fewer than 5 subjects in a group. Descriptive statistics are reported as mean ± standard deviation or frequency and %. A p-value of <0.05 was considered significant. SAS version 9.3 was used to perform statistical tests.

## Results

**Figure 1:**
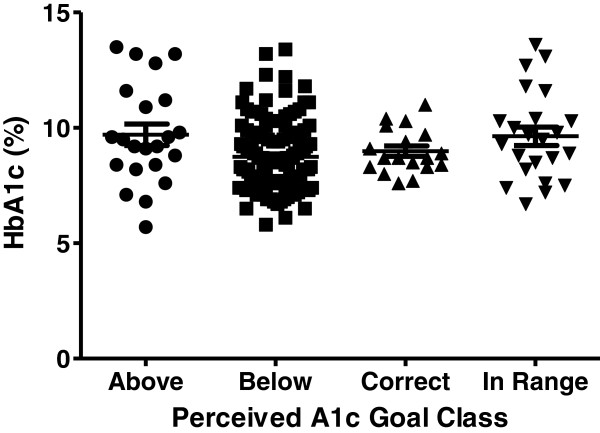
**Actual A1c by Target A1c Category.** Actual A1c values are shown by perceived A1c goal class. ● - subjects whose perceived A1c goal was above the actual target. ■ - subjects whose perceived A1c goal was below the actual target. ▲ - subjects whose perceived A1c goal was correct. ▼ - subjects whose perceived A1c goal was a range that included the actual target.

Of the 240 adolescent subjects with T1D who were surveyed, 52% were male, 78% were non-Hispanic white (11% were Hispanic, 4.2% were black, 1.3% were American Indian or Alaska Native, 0.8% were Hawaiian or Pacific Islander, 0.4% were Asian, and 3.8% were mixed race), mean age was 16.1 ± 1.8 years, diabetes duration was 9.0 ± 3.1 years (range 5.0–17.8 years), and mean A1c was 9.0% ± 1.7% (range 5.7%–14%). Clinical characteristics stratified by A1c below or above target are shown in Table 1. A graph of the actual A1c values by target A1c category is shown in Figure 1. There were 42 subjects (18%) with A1c below target and 198 subjects (82%) with A1c above target.

**Table 1:** Characteristics of subjects with A1c below target compared to those with A1c above target

		**A1c below target**	**A1c above target**	**P-value**
		**(N = 42)**	**(N = 198)**	
Age (years)		15.9 ± 1.8	16.1 ± 1.8	0.33
Hemoglobin A1c,%		7.0 ± 0.5	9.5 ± 1.5	< 0.0001
Sex, n (% male)		24 (57.1%)	101 (51.0%)	0.70
BMI z-score		0.56 ± 0.71	0.70 ± 0.84	0.62
Ethnicity:				0.03
	Non-Hispanic White	39 (92.9%)	149 (75.3%)	
	Hispanic	3 (7.1%)	36 (18.2%)	
	Other	0 (0%)	13 (6.6%)	
Insulin pump use		28 (67%)	109 (55%)	0.16
Categorization:				
	Stated Target Correct	0 (0%)	18 (9.1%)	0.05*
	Stated Target Incorrect	42 (100%)	180 (90.9%)	
Stated Target Above vs. Other		3 (7.1%)	20 (10.1%)	0.24*
Stated Target Below vs. Other		31 (73.8%)	117 (59.1%)	0.07
Stated Target Included vs. Other		4 (9.5%)	19 (9.6%)	0.99*
No Stated Target Given vs. Other		4 (9.5%)	24 (12.1%)	0.79*
Systolic BP (mm Hg)		114 ± 9	115 ± 9	0.56
Diastolic BP (mm Hg)		67 ± 6	70 ± 7	0.02
Total Cholesterol (mg/dl)		144 ± 25	160 ± 38	0.007
LDL-c (mg/dl)		81 ± 21	91 ± 29	0.04
HDL-c (mg/dl)		48 ± 8	51 ± 11	0.50
Triglycerides (mg/dl)		70 ± 28	90 ± 53	0.02
Tanner Stage Pubic Hair:				0.76
	1–3	5 (11.9%)	20 (10.1%)	
	4–5	31 (73.8%)	155 (78.3%)	
	Not assessed by provider	6 (14.3%)	23 (11.6%)	

*Fisher’s Exact.

Almost all subjects (98%, n = 237) reported that they had been told their A1c target by a healthcare provider. Most of those (88%, n = 212) claimed to know their A1c target. Of the 212, only a small portion (8%, n = 18) were correct, 11% stated a target above the recommended target, and 70% stated a target below the recommended target. There were 42 subjects that stated the A1c target as a range, of which 18 (43%) gave a range that was completely below the correct A1c target (e.g. 6–7%) and are included in the below target group, 1 (2%) gave a range that was completely above the correct A1c target (e.g. 8–10%) and is included in the above target group, and 23 (55%) gave a range that included the correct A1c target (e.g. 7–8%) and were analyzed separately.

Mean measured A1c in the 42 subjects below the ISPAD target (<7.5%) was significantly lower compared to the 198 subjects with A1c above target (7.0% vs. 9.5%, p < 0.0001). Among subjects with A1c below 7.5%, 74% thought their A1c target was lower than the actual target, compared to 59% of subjects above target (p = 0.07). Interestingly, among subjects with A1c below 7.5%, none stated the correct A1c target, whereas 18 subjects (9%) with an A1c above 7.5% knew the correct A1c target. Age at diabetes diagnosis had no effect on perceived A1c target (p = 0.23).

Those subjects with an insulin pump (n = 137) had an average A1c of 8.6% ± 1.3%, whereas those using insulin by injection (n = 103) had an average A1c of 9.7% ± 1.9% (p = <0.0001). Of the 137 subjects using an insulin pump, 28 had an A1c below 7.5%, compared to 14 of the 103 using insulin by injection (20% vs. 14%, p = 0.16). Subjects who achieved A1c levels below 7.5% had lower total cholesterol, LDL-c, triglycerides, and diastolic blood pressure (p < 0.05 for all). There was no difference in distribution by Tanner stage among those with A1c below target versus those with A1c above target (Table 1, p = 0.76).

## Discussion

In this cohort of adolescents with T1D, the mean A1c of 9.0% was significantly higher than the ISPAD goal of less than 7.5%. This is similar to glycemic control observed in the T1D Exchange adolescent cohort (mean A1c 8.8%) and slightly higher than a cohort of adolescents with T1D in the SEARCH for Diabetes in Youth study (mean A1c 8.3%) [8,9]. We also found lower cardiovascular disease risk factors (total cholesterol, LDL-c, triglycerides, and diastolic blood pressure) in those subjects with A1c below target compared to those with A1c above target, which is also consistent with the SEARCH adolescent cohort [10,11].

In this cohort, nearly all subjects claimed to know their A1c target, but few were able to accurately identify the A1c target of less than 7.5%. These data suggest that few adolescents with T1D for 5 years or more are aware of their A1c target, despite having diabetes for a mean of almost 10 years. Furthermore, the adolescents in this cohort are all cared for at a large diabetes specialty center by a team including a pediatric endocrinologist and diabetes educators, where diabetes education and A1c goals are reviewed frequently. One would expect these subjects to have a better understanding of diabetes than other adolescents not seen at a diabetes specialty clinic. There was a large number of pateints on insulin pumps, suggesting a higher level of sophisticated diabetes care. However, subjects on insulin pumps gave similar incorrect answers as those adolescents treated with insulin injections.

Among those who believed their A1c goal was lower than the actual A1c target, more subjects had A1c below target than above target. The general trend suggests that those who perceived their A1c goal to be lower than the actual target were more likely to achieve an A1c below the actual target, although this difference did not reach statistical significance. While causality cannot be confirmed from our data, it would follow logically that adolescents who perceive their A1c goal to be lower than the actual target are more likely to strive to achieve that lower goal.

Thus, lowering the A1c target for adolescents could potentially lead to improved A1c levels and decreased long-term complications of diabetes. However, lowering the A1c target could also lead to an increased frequency of hypoglycemia. Data from the Diabetes Control and Complications Trial (DCCT) suggested that more intensive therapy led to significantly higher rates of severe hypoglycemia [3]. Although the DCCT was carried out before the availability of current insulin analogs, the risk for more frequent hypoglycemia should be considered when determining A1c targets for this age group. It is also possible that lowering the A1c target could lead to greater disappointment at not achieving that goal, which could result in unanticipated anxiety and frustration in adolescents with type 1 diabetes. However, evidence regarding adolescent reactions to A1c goals is lacking.

ISPAD has set an A1c target of less than 7.5% for children and adolescents with T1D [4]. This recommendation is based on adolescent and adult data from the DCCT, which showed that an elevated A1c was associated with increased risk for development of microvascular and macrovascular complications and that a lower A1c was associated with increased hypoglycemia [1-3]. The ISPAD A1c target of less than 7.5% represents a balance between decreasing long-term complications of diabetes and minimizing acute episodes of hypoglycemia.

An adult study has shown that when patients know their A1c, they are more likely to report better diabetes care [12]. Similarly, a study in adolescents showed that setting glycemic goals has a strong influence on A1c outcomes [6]. However, another study in children and adolescents has shown that there is a significant lack of knowledge concerning the A1c test and the long-term complications of an elevated A1c [5]. Our data are consistent with these studies in that few adolescents (8%) correctly identified their A1c target.

One of the main limitations of our study is that 42 subjects stated their A1c target as a range, rather than identifying a discrete value. Of those, 23 subjects stated an A1c target range that included the true A1c target of less than 7.5%, many of whom stated their A1c target as 7–8%. If they had been required to give a discrete value as an A1c target, it is unclear if they would have stated less than 7%, 7.5%, or 8%. Thus, their responses required separate analysis, rather than contributing to the analysis of the entire cohort. Another limitation of our study is that we do not have subject-specific data on what education subjects received at each clinic visit. It is possible that some providers may have suggested a higher A1c goal for certain adolescents to avoid hypoglycemia or as a step towards better glycemic control for those in poor control. While we do not have recorded data on how many study participants were given interim targets to pursue improved glycemic control, we do know that all patients were seen at a specialized diabetes clinic where the recommended goals are generally considered to be standard of care and are included in teaching materials provided to all families [13]. An additional limitation of our study is that some subjects may have been seen for a study visit shortly after turning age 13 years, but had not yet been seen at the diabetes clinic to discuss their A1c target.

The prevalence of T1D is increasing in the U.S., and adolescents make up a significant portion of those with T1D [14,15]. Adolescence is a time of cognitive development and increasing autonomy. As adolescents become more independent and more mature, they begin to take more responsibility for the management of their diabetes. This transfer of responsibility often occurs in the early teenage years, and there are a number of factors that can influence their ability to manage their diabetes. One important factor that may influence how well adolescents manage their diabetes is an awareness of treatment goals. Our data suggest that adolescents who think their A1c goal is lower than the ISPAD target may be more likely to achieve a lower A1c.

## Conclusions

In this cohort of adolescents with T1D, we found a general trend that a lower perceived A1c goal was associated with a lower achieved A1c. As adolescents become more responsible for their diabetes care, a discussion of treatment goals should be a consideration. It is important for them to know their A1c target and understand how their actual A1c compares to that target. Further studies should focus on identification of other factors influencing an adolescent’s ability to achieve a lower A1c without excessive hypoglycemia.

## Abbreviations

A1c: Hemoglobin A1c; DCCT: Diabetes Control and Complications Trial; HDL-c: high-density lipoprotein cholesterol; ISPAD: International Society for Pediatric and Adolescent Diabetes; LDL-c: Low-density lipoprotein cholesterol; T1D: Type 1 diabetes.

## Competing interests

The authors declare that they have no competing interests.

## Authors’ contributions

SAC compiled the data and wrote the manuscript. MDA helped compile the data and write the manuscript. FKB collected the data, compiled the data, and edited the manuscript. KKM analyzed the data and performed the statistical analysis. GJK participated in the design of the study and edited the manuscript. DMM participated in the design of the study, collected the data, and edited the manuscript. RPW participated in the design of the study, compiled the data, analyzed the data, and edited the manuscript. All authors read and approved the final manuscript.
